# “Understanding the impact of sensory loss on elders with cognitive impairment: A qualitative study of family carers’ views on implications for quality of life and care in India”

**DOI:** 10.1002/alz70858_106383

**Published:** 2025-12-26

**Authors:** Keerthana Umapathy, Lynne Lohfeld, Suvarna Alladi, Priya Treesa Thomas, Nathan Congdon

**Affiliations:** ^1^ Queen's University Belfast, Belfast, Northern Ireland, United Kingdom; ^2^ National Institute of Mental Health and Neurosciences [NIMHANS], Bengaluru, Karnataka, India; ^3^ Queens University Belfast, Belfast, United Kingdom; ^4^ Sun Yat‐Sen University, Guangzhou, China; ^5^ Orbis International, New York, NY, USA

## Abstract

**Background:**

Impaired vision and hearing (sensory impairment) are common among elders, particularly those with cognitive impairment or dementia. The resulting difficulties with communication, daily functioning and social interactions reduce quality of life for affected persons and their families. Despite recent focus on providing sensory care for elders, this is often a neglected issue due to stigma, limited resources, and logistical barriers particularly in low‐and middle‐income countries. The COVID‐19 pandemic further reduced access to sensory care services, creating additional barriers for community‐dwelling elders and their carers. This qualitative study examined the impact of sensory impairment in elders with cognitive impairment or dementia in India from the family carer perspective, with a focus on how it affects quality of life, care provision as well as barriers to obtaining sensory care.

**Method:**

Data were collected in focus group discussions with family carers of elders with cognitive impairment or dementia. Participants had a wide range of educational and socioeconomic backgrounds, ensuring both depth and breadth in the data. Inductive qualitative content analysis was conducted to derive themes related to carers’ perceptions, experiences and recommendations.

**Result:**

Three focus groups with 13 carers were interviewed in 2023/24. Five themes (ageing, sensory functions, elder care, quality of life and the COVID‐19 pandemic) highlight the issues faced by the elders and their carers due to impaired social interactions and communication, difficulties adapting to sensory loss, as well as barriers to accessing sensory care and services during and after COVID‐19. Participants also provided suggestions on how to increase public awareness and service provision for this segment of the older population.

**Conclusion:**

Addressing vision and hearing loss is vital to ensure the quality of life for elders, particularly those with cognitive impairment or dementia, and their families. Findings highlight the need for community‐based care models and targeted interventions to address gaps in sensory care services for these elders, especially during public health crises.

